# Identification of Post-Ictal Generalised EEG Suppression with Two-Channel EEG

**DOI:** 10.3390/s25164932

**Published:** 2025-08-09

**Authors:** Joe Davies, Ali Zarei, Jonas Duun-Henriksen, Pedro Viana, Sándor Beniczky, Mark P. Richardson

**Affiliations:** 1 Basic and Clinical Neuroscience, King’s College London, London SE1 7EH, UK; pedro.viana@kcl.ac.uk (P.V.); mark.richardson@kcl.ac.uk (M.P.R.); 2 UNEEG Medical A/S, 3450 Allerod, Denmark; a.zarei91@gmail.com (A.Z.); jdh@uneeg.com (J.D.-H.); 3 Clinical Neurophysiology, Danish Epilepsy Centre, 4293 Dianalund, Denmark; sbz@filadelfia.dk

**Keywords:** PGES, SUDEP, subcutaneous, ultra-long-term, machine learning, anomaly detection, VAE, detection, duration

## Abstract

**Highlights:**

**What are the main findings?**

- PGES can be identified using subcutaneous, two-channel EEG.
- Anomaly detection can isolate PGES from background EEG.

**What is the implication of the main finding?**

- Ultra-long-term subcutaneous EEG is a valuable tool for monitoring epilepsy.
- Less invasive techniques for monitoring PGES could help the understanding of SUDEP.

**Abstract:**

This study investigates the feasibility of using a two-channel subcutaneous EEG device (SubQ) to detect and monitor PGES. The SubQ device, developed by UNEEG Medical A/S, offers a minimally invasive alternative to scalp EEG, enabling ultra-long-term monitoring and remote data analysis. We used annotated scalp EEG data and data from the SubQ device. The pre-processing pipeline included channel reduction, resampling, filtering, and feature extraction. A Variational Auto-Encoder (VAE) was employed for anomaly detection, trained to identify PGES instances, and post-processing was applied to predict their duration. The VAE achieved a 100% detection rate for PGES in both scalp and SubQ datasets. However, the predicted durations had an average offset of 35.67 s for scalp EEG and 26.42 s for SubQ data. The model’s false positive rate (FPR) was 59% for scalp EEG and 56% for SubQ data, indicating a need for further refinement to reduce false alarms. This study demonstrates the potential of subcutaneous EEG as a valuable tool in the study of epilepsy and the monitoring of PGES, ultimately contributing to a better understanding and management of SUDEP risk.

## 1. Introduction

Epilepsy is a common neurological disorder affecting approximately 50 million people worldwide [1]. It is a chronic brain condition, defined by recurring unprovoked seizures. Seizures can, in general, be classified into 3 types of onset [2]:

- Focal: those that begin on one side of the brain;
- Generalised: those that being on both sides of the brain;
- Unknown: those where the onset is unclear.

Seizures are characterised by a wide variety of motor and non-motor features, including change in level of consciousness, muscle jerking, abnormal limb postures, semi-purposeful movements (“automatisms”), and changes in autonomic function such as alteration in heart rate [2].

Sudden Unexpected Death in Epilepsy (SUDEP) is a major cause of death in people with epilepsy, particularly those with more frequent generalised tonic-clonic seizures (GTCS) (categorised by loss of consciousness, muscle stiffness, and muscle jerking) which can increase the risk of SUDEP by up to 27 times [3]. The causes of SUDEP are not fully established and whilst some risk factors associated with it are known (GTCS occurrence and living alone [3], for example), some factors are not consistent. For instance, whilst evidence suggests that seizure frequency is the most strongly associated risk factor [4], there are reports of people succumbing to SUDEP during or after their second seizure [5]. The overall incidence rate of SUDEP is approximately 1.2 per 1000 person-years [6], so understanding SUDEP risks and causes is vital to reducing the risk of death for those living with epilepsy.

Electroencephalography (EEG) is a non-invasive technique of measuring the electrical activity of the brain by measuring the voltage fluctuations across electrodes attached to the scalp. Recordings of EEG can be analysed to identify normal physiological brain activities and abnormalities associated with epilepsy, such as interictal epileptiform discharges and seizures. Electrodes are typically placed according to the international 10–20 system [7].

This electrode placement gives full coverage of the brain, is low-cost, and provides excellent temporal resolution. However, signals can be smeared by skull-connectivity effects and, whilst non-invasive, scalp EEG is not practical for more than a few days of monitoring of EEG. This is crucial for providing accurate information about seizure occurrences in patients with epilepsy (PWE) and, by extension, SUDEP.

**Figure 1:**
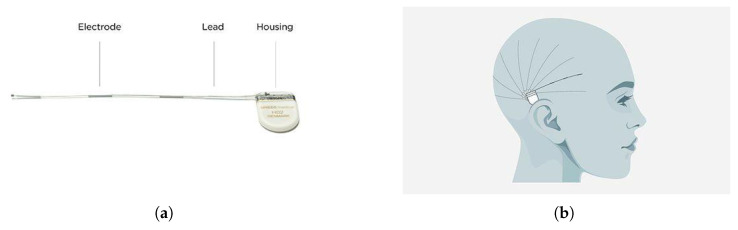
(**a**) The UNEEG SubQ chip and (**b**) possible placements of the device. This is a two-channel device with three electrodes, one used for reference. The placement of the device is either on the right or left side of the brain, with a limited number of channels available.

A novel alternative to scalp EEG is the subcutaneous device from UNEEG Medical A/S [8], (SubQ). Unlike scalp methods, this device offers a minimally invasive alternative by implanting 3 electrodes under the skin, supplying two-channel recordings (the middle being used as a reference). The device and its possible placement can be seen in Figure 1. The device is designed for ultra-long-term use, and data is uploaded by the user to the cloud and automatically analysed. This allows for remote monitoring by healthcare professionals, enabling them to track the patient’s condition over time and make interventions when needed, such as adjusting medication.

Post-Ictal Generalised EEG Suppression is defined as the immediate post-ictal (within 30 s after a seizure) generalised absence of electroencephalographic activity <10µV in amplitude, allowing for muscle, movement, breathing, and electrode artefacts [9]. Whilst PGES does not occur exclusively in cases of SUDEP, all documented cases of SUDEP found some instance of PGES during medical monitoring ([9,10,11,12,13,14]). In addition, it has been reported that instances of PGES lasting longer than 50 s increases the risk of SUDEP, with risk being quadrupled over 80 s [9]. There is not only a link between PGES and SUDEP, but also between PGES and GTCS, with having more than three GTCS in a single year being heavily associated with PGES (p<0.001) [15]. As a result, PGES detection and duration after a GTCS represents a key avenue of exploration in understanding the risk associated with SUDEP.

This research intends to answer whether two-channel EEG is sufficient to allow PGES detection and duration monitoring using data collected from the SubQ device as well as historic scalp EEG. Given the utility of such a device, understanding whether PGES can be detected by the focal placement of the subcutaneous electrodes could be critical in giving patients and clinicians an insight into the risk of SUDEP to the user. We use annotated EEG as well as common and novel statistical techniques to accomplish this, providing a benchmark for future analyses into this topic.

Previous attempts to provide automated detection of PGES have been performed. Both Li et al. [16] and Kim et al. [17] both use random forest methods to achieve their best results. The former finds accuracy scores between 95–97% depending on the prevalence of artefacts in the data. They used a total of eight channels of scalp EEG from a total of 116 recordings from 84 patients. Y. Kim et al. [17] use 10 channels of scalp EEG from a total of 168 patients with one session each. The best result was an accuracy of 83%. Finally, Theeranaew et al. [18] use an ensemble of logistic regression methods with adaptive boosting. They use a bipolar 1020 montage (with no explicit number of channels) and a total of 34 seizures from 32 patients with and 80:20 split for training and testing. Whilst the work does not perform a specific accuracy analysis, they do find that the algorithm performs at approximately the same accuracy as a human clinician.

Most recently, a study by Li et al. [19] introduced a hybrid approach for automated detection of PGES, combining unsupervised K-means clustering based on artifact features with supervised sample-weighted random forest classifiers. Evaluated on a dataset of 268 EEG recordings from 171 patients, the approach achieved a 10 s tolerance-based detection accuracy of 79.85% and an average PGES prediction offset error of 8.26 s.

All of these methods use a mixture of time, frequency, and signal shape features, as well as pre-processing steps that involve Butterworth filters of varying degree as well as notch filters of between 50–60 Hz to remove electrical noise. Crucially, these studies use multiple channels of scalp recording, rather than just the two channels that are available to the SubQ device. This study differs in trying to determine whether two-channel EEG is sufficient for the monitoring of PGES.

## 2. Materials and Methods

### 2.1. Data Overview

The dataset used for this study is comprised of scalp EEG data from 21 people with epilepsy, with a total of 52 seizure sessions recorded [20]. Each scalp EEG recording was comprised of 23 channels (FP1, FP2, F3, F4, C3, C4, P3, P4, O1, O2, F7, F8, P7, P8, CZ, FZ, PZ, F9, F10, T9, T10, P9, P10) using an average reference. Sessions with possible PGES were reviewed by two independent neurophysiologists for PGES and PGES duration. In total, 38 PGES sessions were annotated with a duration of 27.1 ± 2.6 s (mean ± std). As well as this, data collected from Temple University [21] were used in order to get more background EEG samples from those who suffered from GTCS. Five subjects with a total of 11 sessions of scalp EEG were included from Temple University. All of these include a GTCS, but no instances of PGES. This gives a total of 26 people with epilepsy and 63 seizure sessions.

These data were compared to 5 sessions of subcutaneous EEG data from 3 patients, collected by UNEEG Medical A/S using the SubQ device. All 5 sessions were also annotated in the same manner as the scalp EEG for PGES and PGES duration, with each session containing at least one instance of PGES.

It should be noted that only background and PGES instances were included in these datasets, meaning seizures were removed before pre-processing. This was done using time stamps as provided by clinical experts.

There is a clear discrepancy in numbers of individuals used for the training and testing, i.e., 26 patients with scalp EEG in comparison to just 3 with SubQ EEG. This reflects the nature of the technology being new, so not much data has been able to be collected and analysed to date for the SubQ device. Ongoing studies using the device [22] aim to collect more data to further refine this work.

### 2.2. Pre-Processing

**Figure 2:**
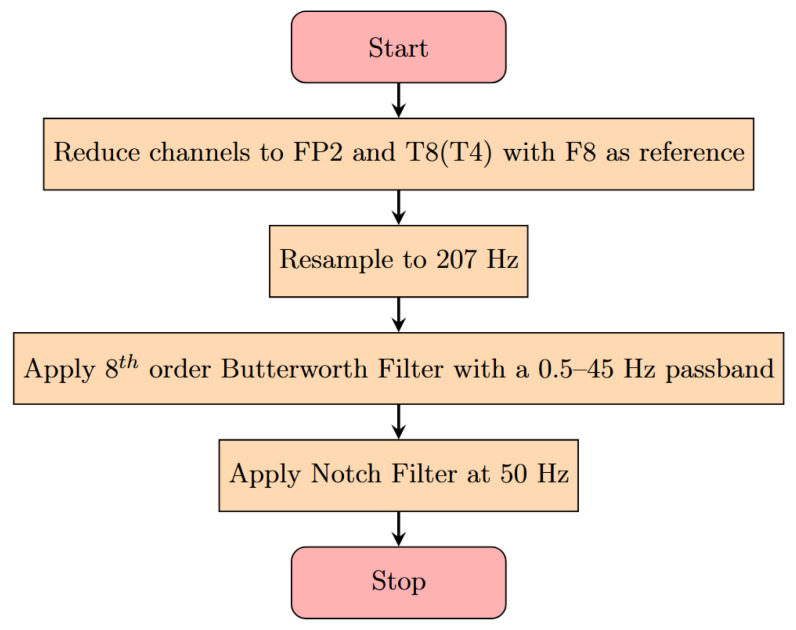
Pre-processing flow diagram for the scalp EEG. Channels are chosen to reflect the most common channels available after implantation of the device. Filters are chosen to remove common artifacts such as muscle movement and electrical equipment noise. The resampling to 207 Hz is performed to reflect the true sampling rate of the SubQ device.

A flowchart for the pre-processing can be seen in Figure 2. First, the number of channels was reduced to the ones that are most similar to those covered by SubQ, FP2, and T8 (aka T4). These were both referenced to F8. The original signal frequency was 1024 Hz, which was down-sampled to 207 Hz to be in-line with the operating frequency of the SubQ device again. After this, an 8th order Butterworth filter [23] was applied using a passband of 0.5–45 Hz, and a 50 Hz notch filter was used in order to handle any filter roll-off and electrical noise effects persisting after the band-pass filter. This was done using functions available in the MNE python library [24]. These pre-processed data were then made into a python dataframe [25] in order to facilitate feature extraction and computation.

### 2.3. Feature Extraction

In total, 14 features were used in the final algorithm, chosen after literature review of other PGES searches ([16,18,20,26]). This was done by first using a sliding window of 5 s with an overlap of 2.5 s, creating data epochs. Then features were calculated per epoch, with each epoch being tagged with either a 0 or 1 to indicate whether PGES was present. Features used were as follows:

- Time Based: the mean, standard deviation, median, maximum, and minimum absolute values of potential difference in each channel for each epoch.
- Frequency Based: the absolute and relative Power Spectral Density (PSD) for the delta (0.5–4 Hz), theta (4–8 Hz), alpha (8–12 Hz), and beta (12–40 Hz) frequency bands in each channel for each epoch.
- Petrosian Fractal Density: a measure of the complexity and irregularity of signals in each channel for each epoch.

### 2.4. Detection Method

The technique used to detect PGES is known as anomaly detection. This looks to identify outliers that vary drastically from a dataset’s ordinary behaviour. This method is often used for processes such as understanding complex medical conditions like clinical emergency prediction [27], cancer data integration [28], and simulation of heterogenous clinical study data [29].

**Figure 3:**
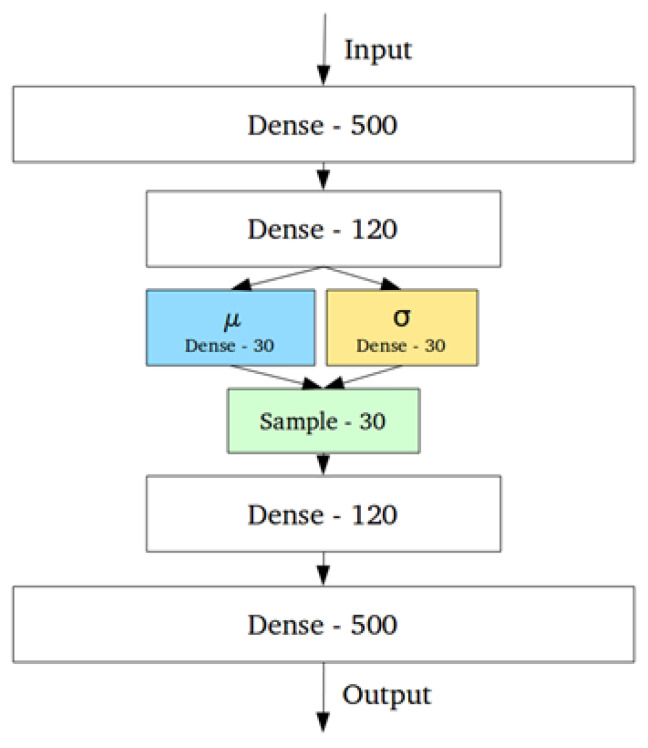
Graphical representation of a Variational Auto-Encoder. The two sets of dense layers after and before the input and output represent the encoder and decoder, respectively. μ and σ are means and standard deviations, respectively. This architecture allows for more control over the latent space of the model, and the sampling layer includes an ϵ parameter that acts as a lagrange multiplier to aid in model improvement.

A common algorithm used for anomaly detection is the Variational Auto-Encoder (VAE). This is a variation of a neural network. Figure 3 shows an example graphical representation of the VAE. A VAE is comprised of three parts: the encoder, the latent space, and a decoder. The encoder maps the inputs to some lower-dimensional latent space where the data is represented by the means μ(x) and standard deviations σ(x) of the latent distribution q(z|x). Instead of directly sampling from the latent space, the VAE employs the reparametrization trick by adding a small-value parameter that samples from a normal distribution ϵ∼N(0,I). This allows the gradients to flow through the sampling process, making improvement of the algorithm via backpropagation [30] possible. The decoder then takes the sampled value and tries to reconstruct the input.

As mentioned, the VAE is improved by backpropagation and minimising a loss function. This loss function is comprised of the reconstruction error (how well the decoder replicates the original input) and the Kullback–Liebler Divergence (a measure of how well the latent distribution replicates the input distribution). The reconstruction error used in this analysis is binary cross-entropy, commonly used for binary classification problems.

The VAE used in this analysis has an input size of 14 neurons, hidden layers of size 14, and a latent space of 2 and built using the PyTorch library [31]. It was optimised using the Adam Optimiser [32]. Activation functions were rectified linear unit (ReLU) for the input and hidden layers, and sigmoid for the output layer, both using pytorch. Batch size and learning rate were 64 and 0.0001, respectively, and the algorithm was trained for 100 epochs. The values mentioned here were chosen through optimisation using an iterative tuning process. This tests a corpus of choices for different hyperparameters (such as neurons in a layer, learning rate, batch size, etc.) by running each combination of parameters and calculating a loss value for each. The VAE with the lowest score is considered the one with the best hyperparameter choices.

Data was split by patients, with a training size of 10, validation of 7, and a test size of 9. The training data was chosen such that a high number of values tagged as background EEG would be included, with a relatively low number of PGES instances. In this way, we move from the classic classification paradigm to anomaly detection.

All data was scaled using the MinMaxScaler function from the scikit-learn python library [33].

**Figure 4:**
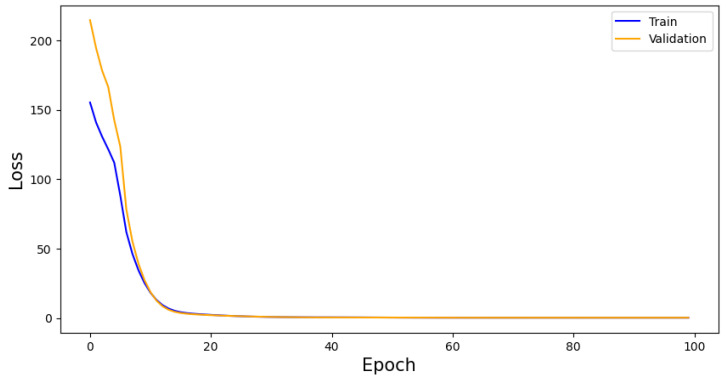
VAE performance as measured by loss per epoch. Whilst the training and validation score begin with a high residual, they converge quickly. This convergences indicates a lack of overtraining, adding to the generalisability of the model.

The algorithm is trained for 100 epochs and monitored for overtraining by considering a loss graph of both training and validation samples. This can be found in Figure 4. The final training and validation losses are 0.195 and 0.219, respectively, showing that overtraining is not an issue for the algorithm.

### 2.5. Post-Processing and Duration

In order to leverage detection to find duration, we use the predicted instances of PGES and background EEG and apply a post-processing method to them. After predictions are made, we are able to count the number of predictions in a row for each session that we know to have PGES. We are then able to compare the predicted PGES length to the truth for each session to get an idea of how good at predicting duration the VAE is per patient. However, there are cases where there may be one or multiple erroneous instances of background or PGES predictions in a long sequence of the other. To avoid this, and so have an improved prediction of duration, a method of flipping misclassified events is employed.

A sliding window of 10 s with a step size of 1 is used across the predictions. The algorithm first tests whether there is a boundary between 0 and 1 (i.e., that the last value in the window is 0 and the next outside the window is 1). Then if 50% or more of the predictions in said window are 0 (background) or 1 (PGES), any instances of the other type are flipped to agree with the majority. The average over all sessions and channels is calculated per patient and the offset from the true value is reported.

## 3. Results

**Figure 5:**
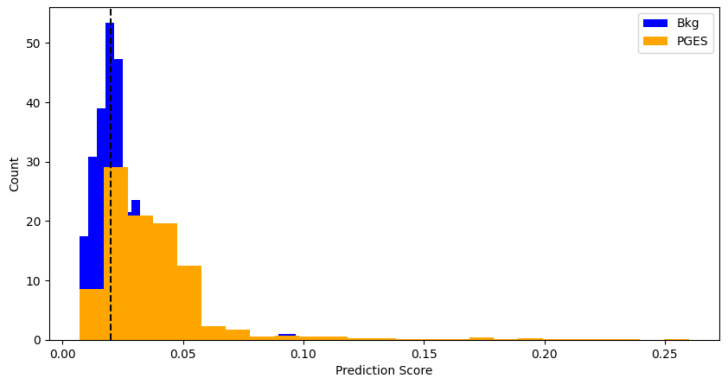
Distributions of predicted scores for background and PGES events in the validation sample. The black dashed line indicates the threshold used for anomaly detection. This threshold is found by repeated prediction-making and testing to see which strikes the best balance between precision and recall.

**Figure 6:**
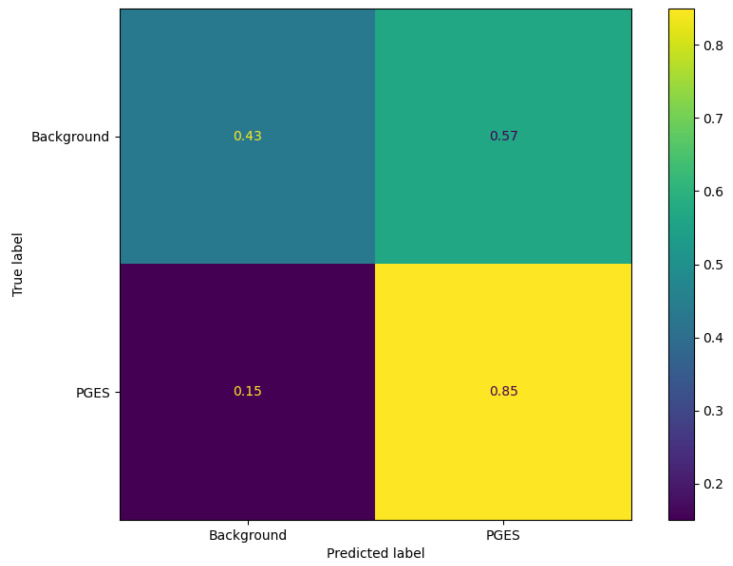
Confusion matrix for the VAE on the test dataset. We can see here that there is a high fraction of correctly identified PGES instances. Background EEG is misclassified often, shown by a higher fraction of background being predicted as PGES.

To get an idea of how well the algorithm is performing, predictions are made using the test data and an anomaly threshold is chosen based on predicted loss scores for the background data and PGES instances present in the validaiton set. The anomaly threshold is chosen to maximise the identification rate of PGES instances in the validation dataset. We first make a set of predictions and then plot the distribution of predicted loss scores for both background and PGES instances. This is seen in Figure 5. We use this to set an anomaly threshold value of 0.02, i.e., any predicted loss value above this will indicate an instance of PGES. Using this, we assign a 0 or 1 to values below or above the threshold and compare those predictions to the true values. In doing so we can compute precision, recall, and F1 scores for the algorithm. Table 1 shows these. Notable here is the difference between the high F1 score for PGES versus the low one for background. We are maximising the identification of PGES over anything else, so this is not particularly important. The high F1 score is a good indication the model is performing well. All instances of PGES in the test (scalp recordings) dataset are found by the algorithm. This is further shown by looking at the confusion matrix for the true and predicted values, shown in Figure 6. Here we notice that the algorithm is very good at identifying PGES, but tends to misclassify background EEG as PGES leading to possible false positives. When using the algorithm on SubQ data, we find that 5 out of 5 instances of PGES are identified as well, giving a 100% detection rate.

The results for the test and SubQ datasets are seen in Table 2 and Table 3, respectively. The average overall offset for the scalp EEG is approximately 36 s. The range being 58 s shows the variability in how well the model and post-processing work together. Notably, patient P22 has an offset of 83.50 s which likely indicates a background EEG pattern similar to that of PGES, or that the PGES instances are not long enough to get an accurate idea of how those instances differ from at-rest EEG, for example.

For the SubQ data we find an average offset that is smaller, approximately 26 s. This, again, arises from a high range of values. The model was not trained on SubQ data, so it is likely here that there is some significant difference between the data collected from scalp EEG versus SubQ arising from the nature of how the data is collected. It is likely that the EEG signal is less noisy being closer to the brain, resulting in PGES and background EEG being more easily distinguished.

In contrast to prior studies that utilize between 8 and 10 scalp EEG channels to achieve high accuracy in automated PGES detection, our work demonstrates that a two-channel EEG setup can yield competitive performance, achieving an F1 score of 82% with an offset error of 32.67 s for scalp EEG. While the offset is higher than the 8.26 s error reported by Li et al. [19] using multi-channel recordings, it is important to note that their method benefits from richer spatial information across more electrodes. Similarly, Kim et al. [17] and Li et al. [16] leverage 8–10 channels to reach accuracies of up to 95–97%, whereas our approach focuses on the practicality and feasibility of using minimal channel configurations, as required by the SubQ device. This trade-off highlights the challenge of capturing complex postictal dynamics with limited spatial coverage, but underscores the potential of streamlined two-channel systems for continuous PGES monitoring outside of clinical environments. Our findings suggest that despite these inherent constraints, two-channel EEG can provide a meaningful and scalable solution for PGES detection, paving the way for more accessible SUDEP risk assessment tools.

## 4. Discussion

In this paper, we present an anomaly detection approach for identifying PGES and predicting its duration using two EEG channels. The VAE detects 100% of PGES instances in SubQ but requires improvement in predicting durations, especially for clinical use. PGES durations above 50 s significantly increase SUDEP risk, with 0–20 s being the least dangerous. Future work should aim to reduce the duration offset to about 10 s.

Detection and duration would require different approaches to effectively improve. Detection would be improved by improving the accuracy of the algorithm, which could be achieved by future studies focusing attention on the latent space representation of the data. It has been shown in other fields that by not assuming a normal distribution, but instead looking at other methods of initialising and manipulating the latent distribution, better accuracies can be achieved [22].

**Figure 7:**
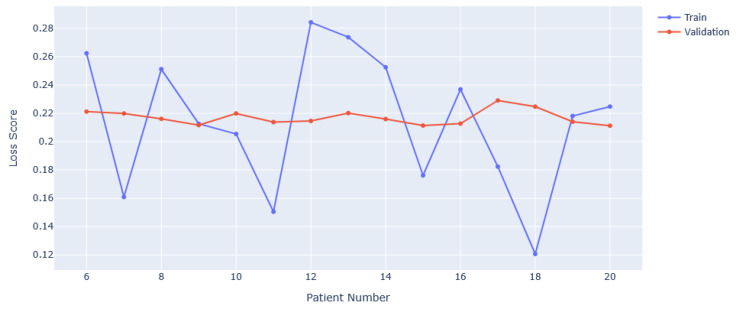
Training and validation loss scores with varying numbers of patients used in training. The minimum is set at 6 as this corresponds to 5 background-only EEGs and 1 background + PGES instance EEG.

Duration improvement relies on the post-processing to be more accurate. This could be achieved by having a more advanced version of the algorithm used to flip misclassified predictions. Instead of using a sliding window with majority rule, an algorithm could be trained to recognise faulty strings or misclassified predictions. This would require a corpus of data with correctly predicted, or labelled, epochs to learn from, with algorithms needing to be able to take variable length inputs. This is possible with long short-term memory (LSTM) type models that compare inputs of different lengths in order to make inferences. In general, collecting more data from those suffering from GCTSs with known instances of PGES would help to better train the algorithm, likely making it less sensitive to the variability between patient EEG responses. Investigating the number of patients used in training against the loss value achieved from the model, we can see the results in Figure 7. It is noticeable that the training loss oscillates around the validation loss, but has significant deviances from it. Also, the validation loss does not change significantly from approximately 0.22. These features indicate that the VAE frequently is prone to over- or undertraining, both problems solved by having more data. In the case of overtraining, more data reduces the risk of spurious correlations being found, giving a more general idea of PGES vs. background EEG. For undertraining, more data gives more examples to learn abstract features from. This increases the discriminating power of the VAE. Whilst it is difficult to accurately predict the number of patients that would be required to fix any such issues, studies using VAEs for datasets such as MNIST [34] use 60,000 training images [35,36], achieving accuracies in the high 90%. With the number of patients and sessions used, we have approximately 30,000 samples, just over 1000 per patient. We can then estimate that up to another 30–40 patients would be needed to achieve those results. This should be not be seen as a rigid figure, however, given that other factors will affect model performance.

Evaluating the algorithm performance with regard to the overall false positive rate, FPR, (also known as the false-alarm rate), we find a relatively high rate of 0.59. This would mean that 59% of the time, the model incorrectly identifies some instance of background EEG as PGES. This high false-alarm rate means that patients could be upset by frequent indications of PGES when there are not any. If we extend this to the SubQ data, the FPR is 56%, which is a mild improvement, but still poor for clinical use. Such a high FPR will negatively impact a patient’s experience, given that it could cause significant stress when none is warranted. Our current threshold was selected to maximise sensitivity to PGES without harming overall accuracy. However, given that different clinical settings may require alternate operating points, we propose that threshold calibration could be dynamically adjusted based on context. In addition, many false positives may arise from other kinds of suppression similar to PGES. As such, further research into different kinds of suppression and the effect these have on accurate PGES analysis may serve to refine this work. Post hoc filtering of these suppression modalities could then reduce the FPR when it comes to PGES.

If this method were to be improved to the point where use would be viable with the SubQ device, it would need to be implemented on the device. This would likely involve using technology such as TinyML [37] with the weights of the algorithm being saved and ported over to a microcontroller compliant with TinyML. This would act as a live warning system in the device, with some warning (red light, vibration, noise) being emitted during a PGES event. Finding the duration of the PGES event would be done after the user has uploaded data to a cloud-based pipeline that the post-processing protocol would be a part of.

We see this work as being a starting point for the integration of novel methods of not only analysing PGES data, but also an indication that subcutaneous EEG could be a useful tool in the study of epilepsy. This is dependent on the continued research into this topic, and the continued testing of the SubQ device in the field. Given the nature of the device, limited channels can be accessed, though it does provide users with much more freedom in getting EEG feedback whilst going about their daily lives. Additionally, looking at the full range of channels reachable by the device and including these into the studies will aid in understanding the usability of the device for PGES monitoring. Refinement of the device is ongoing, and, as it gets more robust, it should be expected that further studies yield more robust results. 

## Figures and Tables

**Table 1:** Values of precision, recall, and F1 for the VAE. The low values on background are unimportant if we are able to correctly predict PGES.

Class	Precision	Recall	F1
Background	0.52	0.42	0.46
PGES	0.75	0.85	0.82

**Table 2:** The predicted and true durations for each patient in the scalp EEG test data. Values here are averaged over all sessions a patient underwent.

Patient ID	Predicted Duration (Seconds)	True Duration (Seconds)	Offset (Seconds)
P15	19.62	38.50	18.88
P20	12.17	39.67	27.50
P22	17.50	101.00	83.50
P23	14.50	59.00	44.50
P2	8.50	13.00	4.50
P27	26.50	29.00	2.50
P29	5.00	0.00	5.00
P2	9.50	47.00	37.50
P9	5.50	67.00	61.50
		Average Offset	35.67

**Table 3:** The predicted and true durations for each patient in the SubQ data. Values here are averaged over all sessions a patient underwent.

Patient ID	Predicted Duration (Seconds)	True Duration (Seconds)	Offset (Seconds)
10	1.50	59.00	57.50
5	17.25	7.50	9.75
15	30.50	20.00	10.50
		Average Offset	25.91

## Data Availability

The data presented in this study are available on request from the corresponding author.

## Abbreviations

The following abbreviations are used in this manuscript:

EEG	Electroencephalogram
VAE	Variational Auto-Encoder
PGES	Post-Ictal EEG Suppression
FPR	False Positive Rate
SUDEP	Sudden Unexpected Death in Epilepsy
GTCS	Generalised Tonic-Clonic Seizure
PSD	Power Spectral Density
LSTM	Long Short-Term Memory
